# The value of partnership in patient-driven as well as in researcher-driven projects

**DOI:** 10.1186/s40900-023-00432-7

**Published:** 2023-04-05

**Authors:** Henning Søndergaard, Malene Deele, Hanne Agerskov, Kirsten Lomborg, Jeanette Finderup

**Affiliations:** 1 Danish Kidney Association, Taastrup, Denmark; 2 Department of Nephrology, Odense University Hospital, Odense, Denmark; 3 Department of Clinical Research, University of Southern Denmark, Odense, Denmark; 4 Department of Clinical Research, Copenhagen University Hospital – Steno Diabetes Center Copenhagen, Herlev, Denmark; 5 Department of Clinical Medicine, University of Copenhagen, Copenhagen, Denmark; 6 Department of Renal Medicine, Aarhus University Hospital, Palle Juul-Jensens Boulevard 99, 8200 Aarhus N, Denmark; 7 Department of Clinical Medicine, Aarhus University, Aarhus, Denmark; 8 Research Centre for Patient Involvement, Aarhus University and Central Denmark Region, Aarhus, Denmark

**Keywords:** Patient and public involvement, Patient partner, Patient engagement, Chronic kidney disease, Diabetes

## Abstract

Patient involvement in health research is rarely driven solely by patients, who could be considered to have the highest degree of investment in such research. In the Kidney Connect project, the patients have been the driving force. This commentary considers the following questions: How did we, as patients, lead the work as the driving force in the project? What went well and what did not go so well from our perspective? How did the project compare with work driven by researchers? We argue that projects driven solely by either patients or researchers each have their own limitations. Projects driven solely by patients have some limitations in their robustness, rigour, and likelihood of publication. Nevertheless, a project driven solely by patients has been able to produce findings that are broadly comparable to a project driven solely by researchers that employed methods ensuring robustness and rigour. We suggest collaboration between patients and researchers also for projects driven by patients.

## Abstract (summary)

Health research often uses patients as either participants or partners. Patients running the research is less common, even though the outcomes might be more important to patients than to anyone else. A medical company started the Kidney Connect project, but invited patients to drive it. The main driving role was planning and conducting data collection and analysis of data for the project. In this commentary, patient representatives describe how they led the project’s work, what went well and what did not go so well for patients. It then compares the project’s results with those from similar work that involved patients as partners but had only researchers in charge. We found that certain things can limit research that is run only by patients, but it has similar results to a project with only researchers in charge.

## Background

In 2015, Novo Nordisk, a large Danish medical company, decided to enhance its research approach by asking the Danish Kidney Association to lead a project investigating patients’ perspectives on better diabetic kidney care. The initiative came from the company, but the project was driven by the patient representatives from the Danish Kidney Association. The project was named Kidney Connect. Novo Nordisk involves patients in medical research and development in six different roles: research subjects, insight providers, advisors, reviewers, co-researchers, and driving force. In the Kidney Connect project, Novo Nordisk wanted the patients to take the role of the driving force [1], which e.g. meant getting a research team together and developing the research protocol. Novo Nordisk’s goal was to improve its understanding of Danish patients’ experiences of diabetic kidney disease at an early stage in order to support decision-making on strategies for outreach to patients to provide the best possible treatment and timely information. The owner of the project was the Danish Kidney Association, with Novo Nordisk acting as sponsor and partner. There is a lack of knowledge about patient driven research and also guidance for patient partners being the driving force. This paper considers the following questions:

- How did we, as patients, lead the work as the driving force in the project?
- What went well and what did not go so well from our perspective?
- How did the project compare with work driven by researchers?

## How did we, as patients, lead the work as the driving force in the project?

Two patient representatives from the Danish Kidney Association were in charge of the entire project, including design, data collection, and data analysis (first and second authors of this paper, hereafter referred to as ‘we’). We were recruited by the Danish Kidney Association, having conducted other projects for the association, although not research. Both of us hold master’s degrees (in psychology and law respectively). We had support for data collection and data analysis from researchers at Novo Nordisk. The scope of the project was decided by Novo Nordisk, which aimed to determine a future-oriented patient vision for diabetes and kidney care and treatment, backed by survey data and voice-of-patient qualitative data. The project used four different methods in pursuit of its aim: 1) a question lab to determine mutually interesting areas of enquiry, 2) a survey to identify overall trends in attitudes and behaviours among patients and to recruit patient participants, 3) in-home semi-structured interviews to gather rich descriptive data to understand underlying motivations, and 4) a future co-visioning workshop to describe and define an ideal future for care and treatment.

**Fig. 1:**
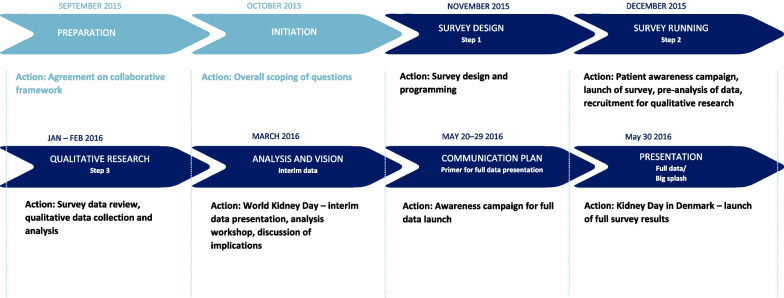
Workflow of the kidney connect project

Figure 1 shows the workflow of the Kidney Connect project.

## What went well and what did not go so well from our perspective?

The initiative for the project came from Novo Nordisk, but as the patients in charge of the project, we had a great deal of freedom to act as its driving force. Novo Nordisk decided on the project’s scope and design, including data collection methods. However, we chose our own questions for the survey, developed the interview guide for the interviews, conducted the individual interviews, and did the data analysis both for the survey and the individual interviews. We perceived ourselves as principal investigators during the project, supported by a co-researcher from Novo Nordisk during data collection and analysis. In our role as the driving force, we experienced only a few minor limitations to our freedom to choose a direction for our research. These were most evident during the process of recruiting participants. We wished to recruit participants with only chronic kidney disease or with diabetes and kidney failure (thus in dialysis). Novo Nordisk, however, wanted less restrictive inclusion criteria regarding participants with diabetes and we therefore agreed to include participants with diabetes and reduced kidney function. Recruitment of participants with both chronic kidney disease and diabetes was consistent with the scope of the project, and the same methods chosen for data collection could be used. Patients with both diabetes and kidney disease were of great interest to the Danish Kidney Association because this group was not represented in our association. An additional goal was to focus more on how to prevent progression of kidney disease, and we wanted to gain knowledge about living with two chronic conditions.

As patients, we elaborated on the purpose of the study in alignment with our understanding of quality of life. Thus, we included five research questions: 1) How do we live a fulfilling life with two diseases? 2) How do we juggle managing our everyday life and our conditions? 3) What do we want to hold on to in life? 4) What can we let go of? and 5) Who decides what interventions are acceptable? The project was regarded as a win-win by both Novo Nordisk and the Danish Kidney Association because it was designed to achieve goals linked to the respective activities and capabilities of both organisations. To be a win-win, a project also needs to have an output. However, at the time of writing, no documentation of the output from the project is available on either organisation’s website. The study had some implicit gains as the results came to characterize and shape the foundation of the Danish Kidney Association's Kidney School Program. This indirect output has not been disseminated in a written format and thus begs the questions: Did neither Novo Nordisk nor the Danish Kidney Association win? Did the organisations achieve sufficient value for their efforts?

We were invited to various conferences and meetings that focused on either diabetes or kidney disease in Denmark and internationally to disseminate our findings. Some of the meetings were held by Novo Nordisk for its own international guests. Between March 2016 and March 2019, we gave a total of eight presentations (four each). One output goal was to write a paper reporting the project results—a short report for either the Danish Kidney Association website or newsletter—but we also wished to publish a paper on the results in a scientific journal. Due to illness and the effects of suffering from a chronic disease, especially fatigue, we did not succeed in this goal. In 2020, a collaboration with a professor in patient involvement (author KL) resulted in a draft of a comment article that we planned to submit to a Danish newspaper. However, again, we did not have the energy at that time to work collaboratively with the researcher to complete the final draft for publication. To finally share our experiences, this article’s team of authors was established in autumn 2022, comprising a mixed group of patients and researchers. The previous lack of a formal scientific paper as an output was not only due to a lack of energy, but also because the study was not conducted with sufficient robustness and rigour. The survey was self-developed without tests of its reliability and validity (even not content and face validity) [2]. The interviews were conducted by interviewers with no experience or training in qualitative interview and data analysis methods, thereby causing a risk of bias. In that the findings could be influenced by our own experiences as patients and not having the skills to work with our subjectivity. In general, we found little guidance on how to conduct patient-driven research of high quality, and this made it difficult to meet the required research standards. Collaboration with researchers with strong methodologic knowledge and the relevant research competences may be the way forward. Being unable to publish a scientific paper, it seemed we had hit an immediate but hidden barrier that would stop us sharing our findings on an equal footing with professional researchers. We had support from researchers, but we did not have a partnership with researchers. A recommendation for the future could be partnership with researchers. Patients being the driving force, do not necessarily mean the patients could not collaborate with researchers and that the patients must be on their own. When researchers establish partnership with patients, it is recommended to clarify expectation from the beginning. When patients establish partnership with researcher, the same should be recommended, that patients clarify expectations with the researchers [3]. Another recommendation could be engaging more patients as the driving force, especially, when the condition may avoid them from working on the project now and then [3].

## How did the kidney connect project compare with work driven by researchers?

**Fig. 2:**
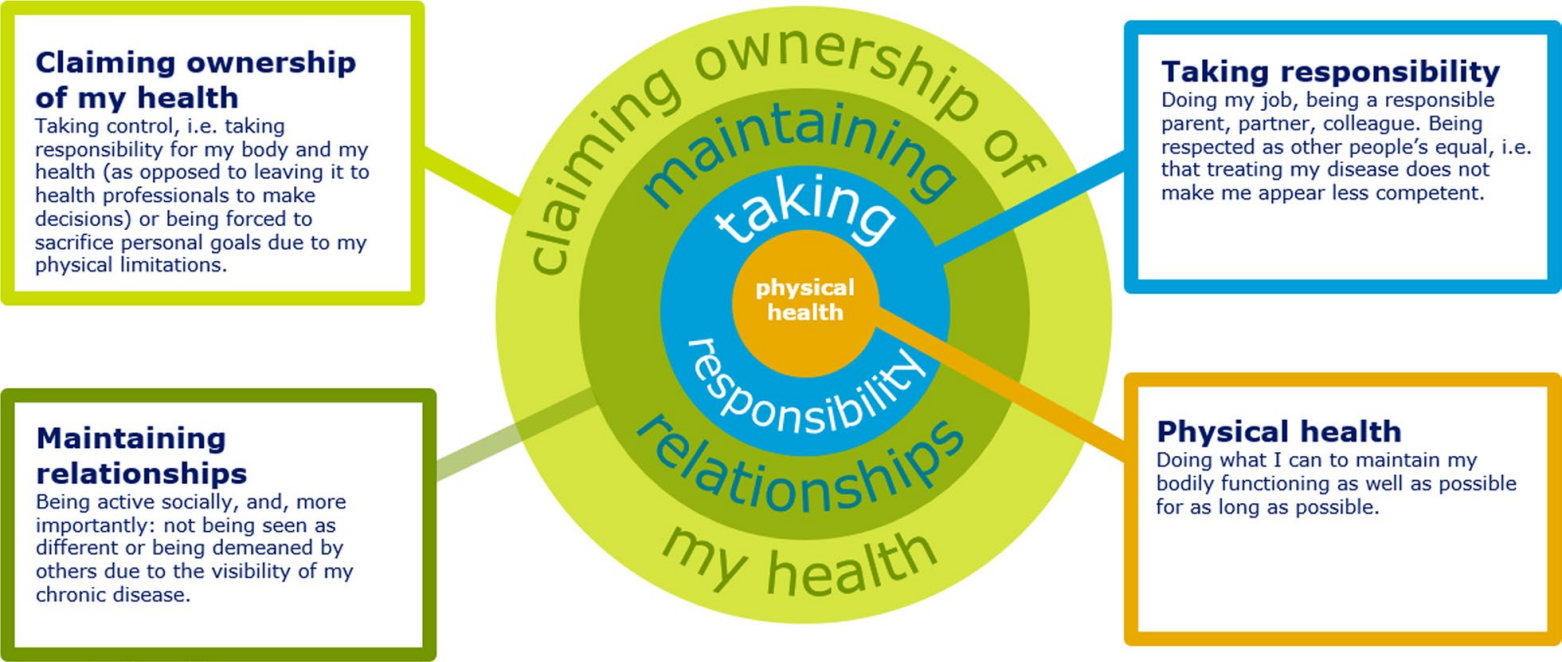
Findings of the kidney connect project

Figure 2 shows the findings of the Kidney Connect project. A literature search in PubMed has shown, that no other studies have explored diabetes and chronic kidney disease patients’ visions of future-oriented diabetes and kidney care and treatment. In general kidney care (i.e. without a specific focus on patients with diabetes), standardised outcomes have been determined through a collaboration between researchers and patients, named the Standardised Outcomes in Nephrology (SONG) Initiative [4]. The SONG Initiative has been driven by researchers, not patients; while patients and relatives have participated, their involvement has been at a lower level [33]. The SONG Initiative has involved five phases: 1) systematic review, 2) nominal group technique with patients and caregivers, 3) stakeholder interviews with patients, caregivers, clinicians, researchers, and policymakers, 4) Delphi survey, and 5) consensus workshop [5]. The methods used in phases 1 and 4 were not part of the Kidney Connect project, and our study included only patients and no other groups of stakeholders. Each SONG Initiative phase has covered different areas of kidney disease and reported different findings. All the findings have subsequently been summed up in a single model showing that living well with kidney disease can be achieved through ‘life participation’. To enhance life participation, symptoms and life impacts must be addressed. Education, engagement, and empowerment must be provided as part of a ‘strengths-based approach’ before clinical strategies are determined [6]. ‘Physical wellness’, ‘sustaining relationships’, and ‘fulfilling responsibilities’ are findings from Kidney Connect which are consistent with ‘address symptoms and life impacts’ from the SONG Initiative. ‘Claiming ownership of my health’ is a finding from Kidney Connect which is consistent with the ‘strengths-based approach’ from the SONG Initiative. As such, all four main findings from Kidney Connect are also to be found in the SONG Initiative’s work. One finding from the SONG Initiative that was not a finding of the Kidney Connect project was ‘clinical strategies’. This is possibly due to the Kidney Connect project having been driven by us as patients ourselves. A Google search in 2023 returned no hits for the Kidney Connect project. In contrast, the SONG Initiative’s website lists more than 50 published research papers, and the synthesis of all its projects produced for World Kidney Day 2021 has been cited in more than 100 journal articles. The Kidney Connect project, was one project, and the SONG initiative includes a lot of different projects, why they are not totally comparable.

## Conclusions

This commentary reports on how patients have been the driving force in a research project using surveys, interviews, and workshops for data collection. Furthermore, patients carried out the data analysis and presented the findings at national and international conferences and meetings. The four main findings in the Kidney Connect revealing the patients' perspective to diabetic and kidney care is claiming ownership of my health, maintaining relationships, taking responsibility and physical health. Projects driven solely by patients have some limitations in their robustness, rigour, and likelihood of publication. Nevertheless, a project driven solely by patients has been able to report findings that are broadly comparable to those of projects driven solely by researchers and that employed methods ensuring robustness and rigour. We suggest, that health research projects driven solely by patients should be transformed to partnerships with researchers, just as projects driven solely by researchers should seek collaboration with patient partners. We suggest to consider the number of patient partners to ensure a robust group of patient partners through the whole project.

## Data Availability

Not applicable.

## Abbreviation

SONG: Standardised outcomes in nephrology
